# Regulation of the phagocytic activity of astrocytes by neuroimmune mediators endogenous to the central nervous system

**DOI:** 10.1371/journal.pone.0289169

**Published:** 2023-07-27

**Authors:** Sijie (Shirley) Yang, Svetlana Simtchouk, Julien Gibon, Andis Klegeris

**Affiliations:** Department of Biology, University of British Columbia Okanagan Campus, University Way, Kelowna, British Columbia, Canada; University of Texas Medical Branch at Galveston, UNITED STATES

## Abstract

The phagocytic activity of glial cells is essential for maintaining normal brain activity, and its dysfunction may contribute to the central nervous system (CNS) pathologies, including neurodegenerative diseases. Phagocytic activity is one of the well-established neuroimmune functions of microglia. Although emerging evidence indicates that astrocytes can also function as CNS phagocytes in humans and rodents, limited information is available about the molecular mechanism regulating this function. To address this knowledge gap, we studied modulation of the phagocytic activity of human U118 MG astrocytic cells and murine primary astrocytes by four CNS inflammatory mediators and bacterial endotoxin lipopolysaccharide (LPS). LPS and cytochrome c (CytC) upregulated, while interferon (IFN)-γ downregulated, phagocytosis of latex beads by human astrocytic cells and phagocytosis of synaptosomes by murine primary astrocytes. Interleukin (IL)-1β and tumor necrosis factor (TNF)-α had no effect on the phagocytic activity of human astrocytic cells but upregulated this function in murine astrocytes. Varying effects of combinations of the above inflammatory mediators were observed in these two cell types. LPS- and CytC-induced phagocytic activity of human astrocytic cells was partially mediated by activation of toll-like receptor 4 (TLR4). By monitoring other functions of astrocytes, we concluded there were no correlations between the effects of the mediators studied on astrocyte phagocytic activity and their secretion of cytokines, cytotoxins, or glutamate. Our study identified four candidate CNS regulators of astrocyte phagocytic activity. Future investigation of molecular mechanisms behind this regulation could identify novel therapeutic targets allowing modulation of this astrocyte-mediated clearance mechanism in CNS pathologies.

## Introduction

Astrocytes are a type of non-neuronal cells in the central nervous system (CNS) that are essential for the maintenance of brain homeostasis (reviewed in [1]). For example, astrocytes participate in energy metabolism, maintenance and modulation of the blood-brain barrier, as well as the formation, function, and elimination of synapses (reviewed in [2–4]). Furthermore, the contribution of astrocytes to neuroinflammatory responses is increasingly recognized (reviewed in [5,6]). Astrocytes have been established as both a source of and target for numerous inflammatory signaling molecules, including cytokines (e.g., interleukin (IL)-1β, IL-6), chemokines (e.g., monocyte chemoattractant protein (MCP)-1, C-X-C motif chemokine ligand (CXCL)12), and growth factors (e.g., glial cell line-derived neurotrophic factor (GDNF), granulocyte-macrophage colony-stimulating factor (GM-CSF)) (reviewed in [5,7,8]) [9–12]. In addition, recent evidence indicates that astrocytes participate in neuroimmune defense and CNS maintenance by phagocytosing tissue debris and abnormal protein aggregates (reviewed in [13]) [14,15].

Phagocytosis, a critical part of the immune response, is the process where cells detect, engulf, and digest large solid particles with a diameter of more than 0.5 μm. It is an active cellular process that involves the formation of actin-based membrane protrusions, followed by engulfment and digestion of the particles (reviewed in [16]). Microglia, the brain-resident macrophages, are well-established phagocytes of the CNS. Microglial phagocytic activity can be both protective and deleterious (reviewed in [17]). On one hand, by removing excess synapses and neuronal precursor cells, microglial phagocytosis plays a vital role in brain development [18], but on the other hand, adverse inflammatory activation of microglia leads to phagocytosis and death of healthy viable neurons [19,20]. Moreover, phagocytic processes of microglia are activated in various CNS pathologies since these cells, for example, engulf amyloid β (Aβ) aggregates that contribute to Alzheimer’s disease pathology [21,22]. Notably, several risk genes associated with this neurodegenerative disorder (e.g., *TREM2*, *SORL1*, *CR1*, and *BIN1*) are known to impact the clearance of tissue debris and pathological protein structures by microglia (reviewed in [23]).

Compared to microglia, the phagocytic activity of astrocytes is significantly less studied and only very limited information about its contributions to the pathophysiological processes of the CNS is available. It has even been suggested that astrocyte phagocytic activity only plays a compensatory role under circumstances when microglia are impaired or absent and cannot fulfill phagocytic functions [24]. Nevertheless, experimental evidence has been presented demonstrating that both human and murine astrocytes, when exposed to damaging stimuli such as bacterial toxins and inflammatory mediators, phagocytose cellular debris [14], apoptotic cells [25], synaptic structures [26], axons and axonal organelles [27,28], myelin debris [29], and abnormally aggregated proteins [15].

While modulators of microglial phagocytic activity have been studied extensively and include a range of pathology-associated molecules (e.g., complement C1q, C3b) [30] and inflammatory mediators (e.g., lipopolysaccharide (LPS), IL-1β, and interferon (IFN)-γ) [31–33], regulation of this function in astrocytes is not well understood. Studies have shown that astrocyte phagocytic activity can be modulated by Aβ [34], α-synuclein preformed fibrils [35], LPS [36], hemoglobin [37], synthetic steroid tibolone [38], glucocorticoids [39], insulin, and ganglioside GM1 [40]. However, regulation of this critical astrocyte function by immune mediators that are endogenous to the CNS is largely unknown except for a single report by Kalmar et al. [36]. They demonstrated that IL-1β upregulated phagocytosis of latex beads by cultured rat primary astrocytes.

To address this knowledge gap, we hypothesized that, similar to microglia, the phagocytic activity of astrocytes is regulated by the CNS inflammatory mediators. We used two different astrocyte model cell types and two phagocytosis substrates. Initially, phagocytosis of a widely used substrate, latex beads, by human U118 MG astrocytic cells was studied [41]. This was followed by more physiologically relevant experiments assessing the uptake of mouse brain-derived synaptosomes by murine primary astrocytes, which ensured species compatibility. We tested the effects of LPS and three other inflammatory mediators known to regulate microglia phagocytic activity: IL-1β, tumor necrosis factor (TNF)-α, and IFN-γ [31,33,42]. In addition, we studied the effect of a mitochondria-derived damage-associated molecular pattern (DAMP) cytochrome c (CytC), which regulates several neuroimmune functions of astrocytes and microglia [43,44]. In addition, due to the emerging evidence that combinations of inflammatory signals effectively induce astrocyte reactivity [45–47], we studied the effects of three different combinations of these inflammatory mediators on astrocyte phagocytic activity. The modulatory activities of the mediators on phagocytic activity were compared with their effects on several other neuroimmune functions of astrocytes, including their secretion of cytotoxins, glutamate, and select cytokines.

## Materials and methods

### Reagents

The following reagents were purchased from Sigma Aldrich (Oakville, ON, Canada): 3-(4,5-dimethyl-2-thiazolyl)-2,5-diphenyl-2H-tetrazolium bromide (MTT), bisbenzimide (Hoechst 33258), β-nicotinamide adenine dinucleotide hydrate (β-NAD), CytC from bovine heart, diaphorase from *Clostridium kluyveri*, Fluoroshield^TM^ with 4’,6-diamidino-2-phenylindole (DAPI), iodonitrotetrazolium chloride, LPS from *Escherichia coli* 055:B5, N-(1-naphthyl)ethylenediamine dihydrochloride, sulfanilamide, and toll-like receptor 4 (TLR4) antagonist TAK-242.

Bovine serum albumin, calf bovine serum (CBS), fetal bovine serum (FBS), HyClone^TM^ Dulbecco’s modified Eagle medium nutrient mixture F-12 (DMEM-F12), and penicillin-streptomycin (with or without amphotericin B) solutions were from Cytiva (Marlborough, MA, USA). cOmplete^TM^ ethylenediaminetetraacetic acid (EDTA)-free protease inhibitor cocktail was from Roche Diagnostic Canada (Laval, QC, Canada). Cytochalasin B (CytoB) was purchased from Cayman Chemical Company (Michigan, USA). Human and murine recombinant IL-1β, TNF-α, IFN-γ as well as enzyme-linked immunosorbent assay (ELISA) kits for human IL-6, human GM-CSF, and human and murine IFN-γ inducible protein-10 (IP-10) were purchased from PeproTech (Cranbury, NJ, USA). CellBrite® Fix 555 was obtained from Biotium (Fremont, CA, USA). Rhodamine-labeled wheat germ agglutinin (WGA) was from Vector Laboratories (Newark, CA, USA). Goat anti-rabbit IgG CY3-labeled antibodies were from Jackson ImmunoResearch (West Grove, PA, USA). Hank’s balanced salt solution (HBSS) was from Wisent Inc. (Saint-Jean-Baptiste, QC, CA). L-glutamate dehydrogenase and rabbit anti-glial fibrillary acidic protein (GFAP) antibody (#Z0334) were purchased from Agilent (Santa Clara, CA, USA). Fluorescein isothiocyanate (FITC) surface-labeled fluorescent polystyrene latex beads (one μm diameter) were obtained from Bangs Laboratories (Fishers, IN, USA).

### Cell culture models

The human U118 MG astrocytic cell line was purchased from the American Type Culture Collection (ATCC, Manassas, VA, USA). Human SH-SY5Y neuroblastoma cells were donated by Dr. R. Ross (Fordham University, Bronx, NY, USA), and the murine NSC-34 neuronal cells were gifted by Dr. A. Milnerwood (Brain Research Centre, UBC, Vancouver, Canada). Cells were cultured in DMEM-F12 supplemented with 10% heat-inactivated CBS or FBS, penicillin (100 U/ml), and streptomycin (100 μg/ml). Cell cultures were incubated at 37°C in a humidified atmosphere containing 5% CO2.

All experimental procedures involving animals were in compliance with the guidelines of the Canadian Council on Animal Care and were approved by the research ethics committee of our university: The University of British Columbia Animal Care Committee, Vancouver, BC, Canada, protocol A20-0105. C57BL/6NCrl mice (Charles River Laboratory, Montreal, Quebec, Canada) were maintained in the animal facility, and housed in sterilized, filter-topped cages. Efforts were made to reduce animal handling and use. Murine primary astrocyte cultures were obtained from postnatal day one to four pups by following a previously published procedure with minor modifications [48,49]. After removing the meninges, cerebral hemispheres were cut into pieces and incubated in HBSS with 0.25% trypsin at 37°C for 20 min. Deoxyribonuclease I (DNase I) was added for 15 min followed by centrifugation of the cell suspension (5 min at 400 g) and removal of the supernatant. The pellet was resuspended vigorously in DMEM-F12 containing 10% FBS, penicillin (100 U/ml), streptomycin (100 μg/ml), and amphotericin B (500 ng/ml) to obtain single-cell suspensions. Cells were plated in poly-D-lysine (PDL) coated T75 flasks and incubated for eight days, with medium refreshed every two days. The flasks were first shaken at 180 rpm for 60 min to detach microglia, which were discarded, followed by shaking at 240 rpm for another six h to remove oligodendrocyte precursor cells. After washing with warm phosphate-buffered saline (PBS) twice, the astrocyte layer was detached by trypsin-EDTA, cells spun down, resuspended, plated in a new T75 culture flask, and incubated in the 37°C incubator with 5% CO_2_.

### Phagocytosis by human U118 MG astrocytic cells

Human U118 MG astrocytic cells were plated in four-chambered glass-bottom Petri dishes at a density of 5 x 10^4^ cells/ml in 500 μl of DMEM-F12 containing 10% CBS and antibiotics and allowed to adhere for 24 h. In selected chambers, cells were incubated with 20 μM TAK-242 or its vehicle solution dimethyl sulfoxide (DMSO) for 30 min prior to adding the following stimulants or their vehicle solution (PBS) for 48 h: LPS (400 ng/ml), IL-1β (150 U/ml), TNF-α (20 ng/ml), IFN-γ (100 U/ml), and CytC (50 μg/ml). The concentrations for all the mediators used in this study were selected based on previous research in this and other laboratories showing their effectiveness on other neuroimmune functions of human astrocytic cells [44,49,50]. Three different combinations of IL-1β, TNF-α, IFN-γ, and CytC at the same respective concentrations were also tested. Three μl of suspension containing one μm diameter FITC surface-labeled latex beads (10 mg/ml) were added to each chamber. Following one h incubation, external beads were washed away and cell membranes were stained by adding rhodamine-labeled WGA (10 μg/ml) for 10 min [51]. Cells were fixed in 500 μl of cold 70% ethanol for five min and bisbenzimide (2 μg/ml) was added to stain the nucleus prior to imaging. In a separate series of experiments, CytoB (10 μM) was added to U118 MG astrocytic cell cultures 30 min before cells were exposed to LPS or CytC in order to block actin polymerization and consequently phagocytosis [16]. Human U118 MG astrocytic cells were imaged at 40x magnification with a Zeiss AxioObserver.Z1 inverted widefield fluorescence microscope with ZEN 2 (Black edition) software. Three channels were used: 350/470 nm for bisbenzimide, 470/520 nm for fluorescent beads, and differential interference contrast (DIC) for brightfield microscopy. Measurements of corrected total cell fluorescence intensities were performed by an investigator blinded to the experimental condition using the NIH ImageJ program (FIJI build). An additional 594/615 nm channel was used when cell membranes were stained with rhodamine-WGA.

### Preparation of synaptosomes

Synaptosomes were prepared according to a previously published protocol with minor modifications [41,52]. Cerebral hemispheres (after removing cerebellum and olfactory bulbs) were dissected from C57BL/6Crl wild-type adult mice and placed in cold lysis buffer (tissue weight: buffer volume = 1:10) containing 0.32 M sucrose, 5 mM HEPES, and cOmplete^TM^ protease inhibitor cocktail. All the subsequent procedures were performed at 4°C. Tissue pieces were transferred to 1.5 ml tubes and centrifuged at 1000 g for 10 min to remove cell nuclei. The supernatant was then centrifuged at 12000 g for 10 min. The pellet containing crude synaptosomes was resuspended in PBS and stained for 15 min at 37°C with CellBrite® Fix 555 or CellBrite® Fix 640 (1:1000). To remove redundant dye, stained synaptosomes were dialyzed overnight in PBS at 4°C. The presence of the synaptic marker postsynaptic density (PSD)-95 in the preparation was confirmed using immunoblotting.

### Phagocytosis by murine primary astrocytes

Murine primary astrocytes were seeded in four-chambered glass-bottom Petri dishes (5 x 10^4^ cells/ml per well) in 500 μl of DMEM-F12 supplemented with 10% FBS and antibiotics. After 24 h, cell culture medium was refreshed, stimulants or their vehicle solution (PBS) added, and the cells were then incubated for additional 48 h. The medium was replaced in the wells, and 10 μl of diluted synaptosomes (1:5 stock) were added. After two h incubation, external synaptosomes were removed by washing twice with PBS, cells were fixed with PBS containing 4% paraformaldehyde and permeabilized in PBS containing 0.1% Triton X-100. Since murine primary astrocyte cultures contain other glial cell types, astrocytes were visualized by immunostaining for GFAP, which is an astrocyte-specific marker. Fixed cells were washed twice with PBS and blocked in PBS containing 2.5% bovine serum albumin and 0.02% Triton X-100 for one h at room temperature. After incubating overnight at 4°C with anti-GFAP antibody (1:2000 dilution in blocking solution), cells were washed three times with PBS. Goat anti-rabbit CY3-labeled antibodies (1:1000) were added for one h at room temperature. Cells were washed three times with PBS, and DAPI or bisbenzimide nuclear stain was added to the wells before imaging. Murine primary astrocytes were visualized at 40x magnification using a Zeiss AxioObserver.Z1 inverted widefield fluorescence microscope and imaged with Zen 2 software using three channels: 350/470 nm for DAPI-stained nuclei, 470/520 nm for GFAP-positive murine primary astrocytes, and 594/615 nm for CellBrite® Fix 555-stained synaptosomes. Measurements of corrected total cell fluorescence intensities were performed by an investigator blinded to the experimental condition using the NIH ImageJ program (FIJI build). The engulfment of CellBrite® Fix 640-stained fluorescent synaptosomes (620/670 nm) was confirmed with a TCS SPE-II confocal microscope (Leica Microsystems, Concord, ON, Canada).

### Cytotoxicity of human U118 MG astrocytic cells towards SH-SY5Y neuronal cells

Human U118 MG astrocytic cells were seeded in a 24-well plate at a density of 2 x 10^5^ cells/ml in one ml of DMEM-F12 containing antibiotics and 10% CBS. After 24 h incubation, the medium in the plate was refreshed and stimulants were added for 48 h. Human SH-SY5Y neuronal cells were seeded in a 24-well plate in 400 μl of DMEM-F12 containing antibiotics and 5% CBS at a density of 4 x 10^5^ cells/ml. Following 24 h incubation, the medium of SH-SY5Y was aspirated, and 400 μl of the U118 MG cell supernatants were transferred to each corresponding well. The remaining U118 MG cell supernatants were collected and used for ELISAs, which were performed according to the instructions provided by the manufacturer (PeproTech). The MTT cell viability assay was performed on the U118 MG cultures at the time of supernatant transfer, and on the SH-SY5Y neuronal cell cultures at the end of a 72 h incubation period with astrocytic cell supernatants.

### Cytotoxicity of murine primary astrocytes towards NSC-34 neuronal cells

These experiments were performed exactly as described in the previous section for human cell cultures, except that both cell types were seeded in 96-well plates. 250 μl of murine primary astrocyte suspension at a density of 5 x 10^4^ cells/ml in DMEM-F12 supplemented with 10% FBS and antibiotics were added to the plates and the murine NSC-34 neuronal cells were plated in 100 μl of DMEM-F12 with antibiotics and 5% CBS at a density of 2 x 10^5^ cells/ml.

### Glutamate measurements

An enzyme assay was used to measure glutamate concentration in astrocytic cell supernatants by following a previously published experimental procedure [53]. Briefly, human U118 MG astrocytic cells were plated in a 24-well plate at 2 x 10^5^ cells/ml in one ml of DMEM-F12 free of phenol-red containing 5% CBS and antibiotics and exposed to various stimuli or their vehicle solution (PBS) for 48 h. Samples containing glutamate at known concentrations in the same cell culture medium were also prepared and used for the calibration curve. Then, 20 μl of cell-free supernatants or glutamate standard solutions were transferred to a 96-well plate. 155 μl of triethanolamine buffer containing 20 mg/ml β-NAD, 1.2 mg/ml iodonitrotetrazolium chloride, and 3 mg/ml diaphorase were added to each well. An initial absorbance measurement was performed at 490 nm to measure the background. 25 μl of L-glutamate dehydrogenase was added to each well to reach the final concentration of 150 U/ml, and absorbance at 490 nm was recorded every five min until it reached the maximum reading. Concentrations of glutamate that was released into the cell culture supernatants were calculated after the absorbance values of the samples containing only cell culture medium were subtracted from all experimental and calibration curve values.

### MTT cell viability assay

MTT (0.5 mg/ml) was added to the cell cultures and plates were incubated at 37°C for one h. After adding an equal volume of SDS/DMF extraction buffer (20% sodium dodecyl sulfate, 50% N,N-dimethylformamide), plates were incubated at 37°C overnight to allow the formazan crystals to fully dissolve. The absorbance at 570 nm was measured and cell viability data presented as percent compared to values obtained from cells incubated in the growth medium only.

### Statistics

Data were analyzed by using either unpaired or paired Student’s t-test followed by Holm-Šídák’s correction for multiple comparisons where applicable. P values less than 0.05 were considered statistically significant.

## Results

### Phagocytic activity of human U118 MG astrocytic cells is differentially regulated by inflammatory mediators

**Fig 1:**
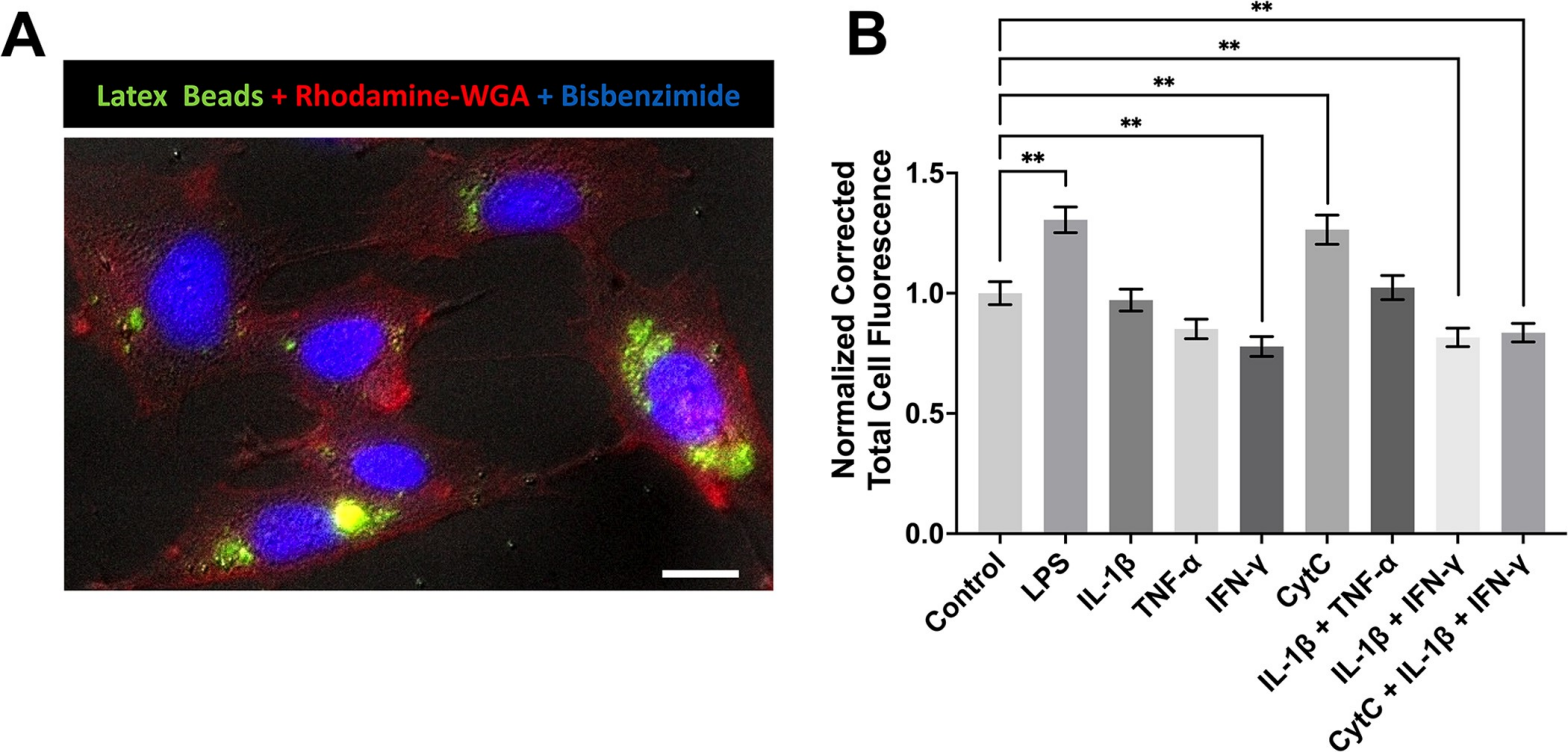
Regulation of the phagocytic activity of human astrocytic cells by immune mediators. A representative DIC-enhanced fluorescent image demonstrating the uptake of latex beads (green) by unstimulated human U118 MG astrocytic cells stained with rhodamine-labeled WGA (red) is shown (A). Cell nuclei are stained with bisbenzimide (blue), and the scale bar represents 20 μm. After 48 h pre-treatment with the mediators shown on the x-axis or their vehicle solution (PBS, Control), human U118 MG astrocytic cells were incubated with latex beads for one h. Fluorescence intensities of 357–825 randomly selected cells were measured. Corrected total cell fluorescence values from six to nine independent experiments are normalized to control values obtained in the absence of immune mediators (B). Data are presented as means ± standard error of the mean (SEM). ** P < 0.01, according to the unpaired Student’s t-test, followed by Holm-Šídák’s correction for multiple comparisons.

**Fig 2:**
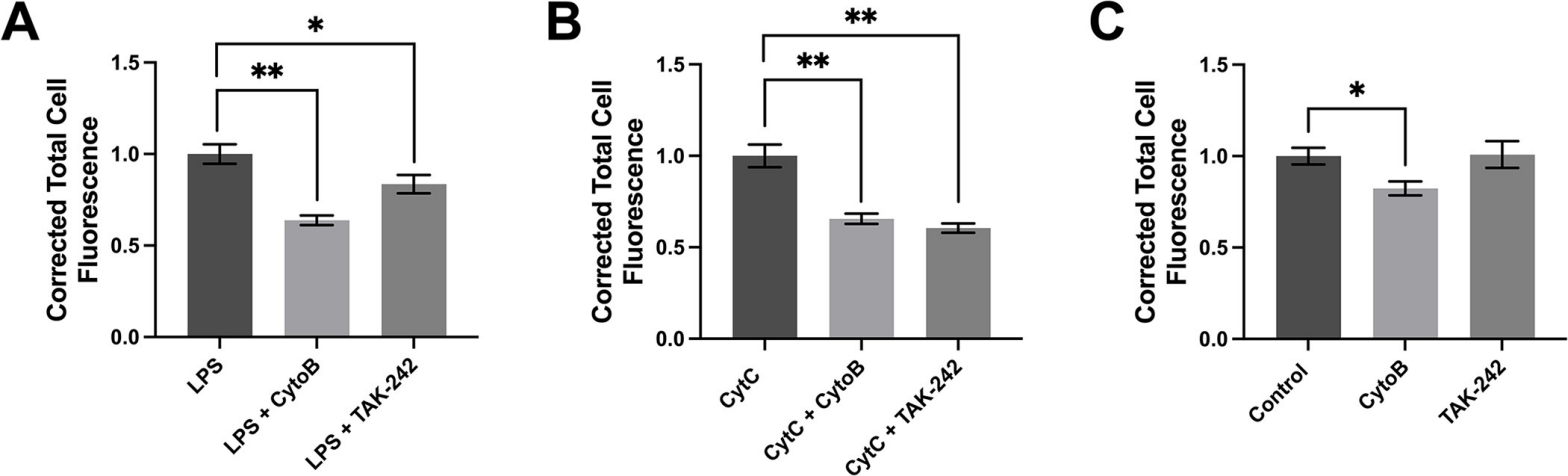
Effects of CytoB and TLR4 inhibitor TAK-242 on phagocytosis of latex beads by unstimulated and LPS- or CytC-stimulated U118 MG astrocytic cells. TAK-242 (20 μM) or its vehicle solution (DMSO) was added to cell cultures for 30 min before their incubation with LPS (400 ng/ml, A), CytC (50 μg/ml, B) or PBS (Control, C) for 48 h. This was followed by the addition of CytoB (10 μM) or its vehicle solution (ethanol) for 30 min, and exposure of cells to fluorescent latex beads for one h. Corrected total cell fluorescence values (means ± SEM) from 188–400 randomly selected cells from five (A, B) or three (C) independent experiments were measured and normalized to values obtained in the absence of CytoB and TAK-242. * P < 0.05, ** P < 0.01, according to the unpaired Student’s t-test, followed by Holm-Šídák’s correction for multiple comparisons.

In the present study, we first sought to identify endogenous modulators of the phagocytic activity of astrocytes. We employed the widely used uptake of fluorescently-labeled latex beads to visualize phagocytosis and human U118 MG astrocytic cells as models of human astrocytes [41]. Fig 1A illustrates the uptake of latex beads by human U118 MG astrocytic cells observed by DIC microscopy. Fig 1B shows that uptake of fluorescently-labeled latex beads by human U118 MG astrocytic cells was significantly modulated by three out of five inflammatory mediators used in this study. The uptake of latex beads was significantly upregulated by LPS and CytC, but downregulated by IFN-γ, as well as the two combinations of cytokines that contained IFN-γ (IL-1β+IFN-γ and CytC+IL-1β+IFN-γ). Meanwhile, IL-1β, TNF-α, and their combination had no effect on the engulfment of beads by U118 MG cells. To confirm that the uptake of latex beads by human astrocytic cells was mediated by phagocytosis, the actin-disrupting agent CytoB was used. Pre-treatment with this inhibitor significantly decreased the uptake of latex beads by LPS- or CytC-stimulated (Fig 2A and 2B), as well as unstimulated (Fig 2C), U118 MG cells, indicating that this process relied on actin polymerization and was thus dependent on phagocytosis.

### TLR4 contributes to the phagocytic activity of human U118 MG astrocytic cells

Previous studies demonstrate that both LPS and CytC interact with the astrocyte and microglial TLR4 [43,44,54,55]; therefore, we hypothesized that this receptor mediated the phagocytic activity of U118 MG cells. Fig 2 demonstrates that a 30 min pre-treatment with a TLR4 inhibitor TAK-242 [44,56] significantly attenuated phagocytosis of latex beads by LPS- and CytC-stimulated U118 MG cells (Fig 2A and 2B) confirming the involvement of TLR4 in the phagocytic activity of these cells. TAK-242 did not affect the phagocytic activity of unstimulated U118 MG cells (Fig 2C).

### Effects of inflammatory mediators on the secretion of cytotoxins, cytokines, and glutamate by human U118 MG astrocytic cells

The inflammatory mediators used in this study have been shown to regulate other immune functions of astrocytes, including their secretion of cytotoxins and inflammatory cytokines (reviewed in [5]); therefore, we explored how the effects of the inflammatory mediators on phagocytic activity related to their functions as modulators of several other well-known functions of astrocytes, including secretion of cytokines and glutamate, as well as cytotoxicity.

**Fig 3:**
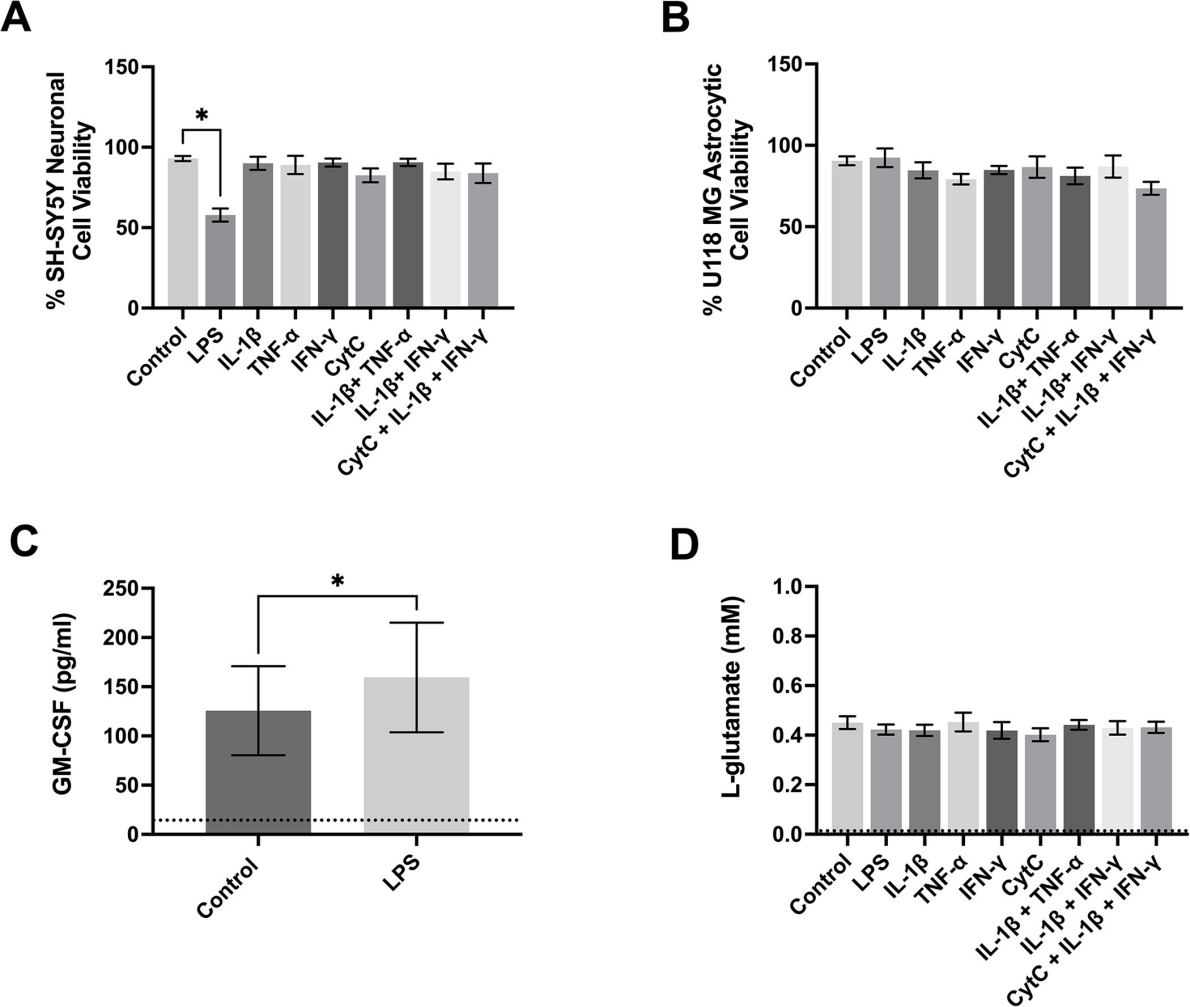
Effects of immune mediators on the human astrocytic cell toxicity towards neuronal cells and their secretion of GM-CSF and L-glutamate. Human U118 MG astrocytic cells were treated with the immune mediators shown on the x-axis or their vehicle solution (PBS, Control), followed by transfer of their supernatants 48 h later to human SH-SY5Y neuronal cell cultures (A, B). The MTT assay was used to determine the viability of astrocytic cells (B) immediately after the transfer of supernatants. Neuronal cell viability was measured by the MTT assay after 72 h incubation with astrocytic cell supernatants (A). An ELISA was used to measure the concentration of GM-CSF (C) in cell culture supernatants, while an enzymatic assay was used to measure the concentration of L-glutamate in supernatants (D). Data from five (A, B) or six (C, D) independent experiments are presented as means ± SEM. The limits of detection for the ELISA and glutamate assay are shown as dotted lines. * P < 0.05, according to the paired Student’s t-test followed by Holm-Šídák’s correction for multiple comparisons (A, C).

Human astrocytes and astrocytic cells have been shown to become toxic towards neuronal cells in response to inflammatory stimulation [57]. Therefore, we used a supernatant transfer assay to determine whether the inflammatory mediators and their combinations we tested in this study induced secretion of cytotoxins by astrocytic cells. Fig 3 demonstrates that out of all the mediators and their combinations tested, only LPS induced a significant toxic effect of human U118 MG astrocytic cell supernatants towards human SH-SY5Y neuronal cells (Fig 3A). None of the stimuli affected the viability of U118 MG astrocytic cells (Fig 3B). None of the stimuli, including LPS, induced secretion of detectable levels of the potential cytotoxin nitric oxide (NO) (S1 Fig).

Reactive human astrocytes secrete a broad range of cytokines and growth factors, including IP-10, IL-6, and GM-CSF [11,44,58]. Secretion of these molecules by human U118 MG astrocytic cells was studied after their exposure to the various inflammatory mediators. IP-10 was not detectable in supernatants of unstimulated U118 MG cells and supernatants from cells exposed to the inflammatory mediators used in this study (S2 Fig). In contrast, U118 MG cells secreted high levels of GM-CSF and IL-6 even in the absence of stimuli. Interestingly, LPS upregulated the secretion of GM-CSF by U118 MG astrocytic cells (Fig 3C), while there were no significant effects of any of the mediators or their combinations on the secretion of IL-6 (S2 Fig).

Astrocyte neurotoxicity can be triggered by attenuated glutamate clearance or active release of this excitotoxic amino acid [59]; therefore, we studied the effects of inflammatory mediators on the concentration of L-glutamate in the supernatants of human U118 MG astrocytic cells. Fig 3D illustrates that L-glutamate concentration was not affected by any of the inflammatory mediators or their combinations tested.

### Murine primary astrocytes phagocytose synaptosomes and this activity is differentially regulated by inflammatory mediators

**Fig 4:**
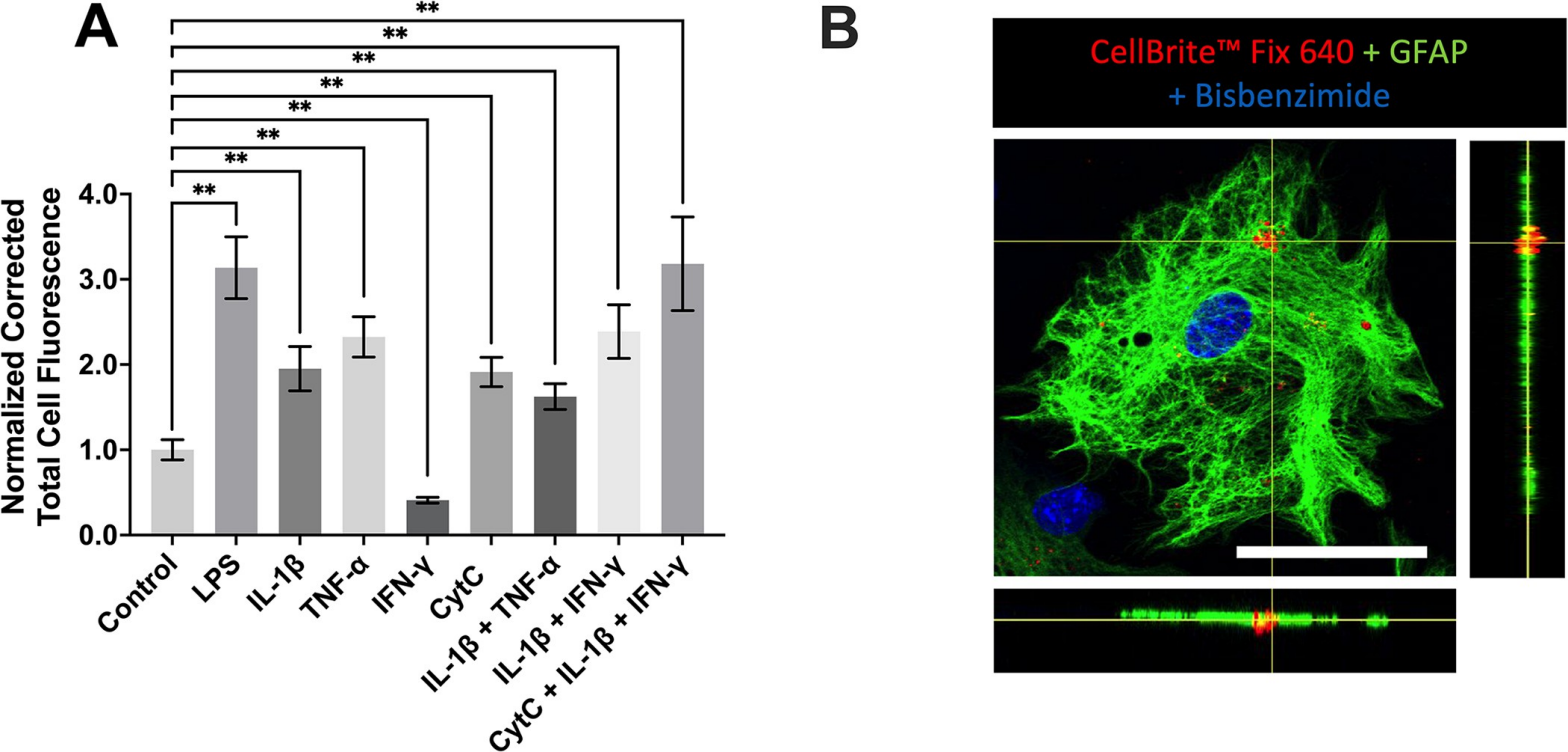
Regulation of murine astrocyte phagocytic activity by immune mediators. After 48 h pre-treatment with the mediators shown on the x-axis or their vehicle solution (PBS, Control), murine primary astrocytes were incubated with murine brain-derived synaptosomes for two h. Fluorescence intensities of 138–216 (A) randomly selected cells were measured. Corrected total cell fluorescence values from three to five independent experiments are normalized to control values obtained in the absence of immune mediators. Data are presented as means ± SEM. ** P < 0.01, according to the unpaired Student’s t-test, followed by Holm-Šídák’s correction for multiple comparisons. A representative confocal image showing the uptake of synaptosomes pre-labeled with CellBrite Fix 640 (red) by GFAP-positive primary murine astrocytes (green) is shown (B). Cell nuclei are stained with bisbenzimide (blue), and the scale bar represents 50 μm.

**Fig 5:**
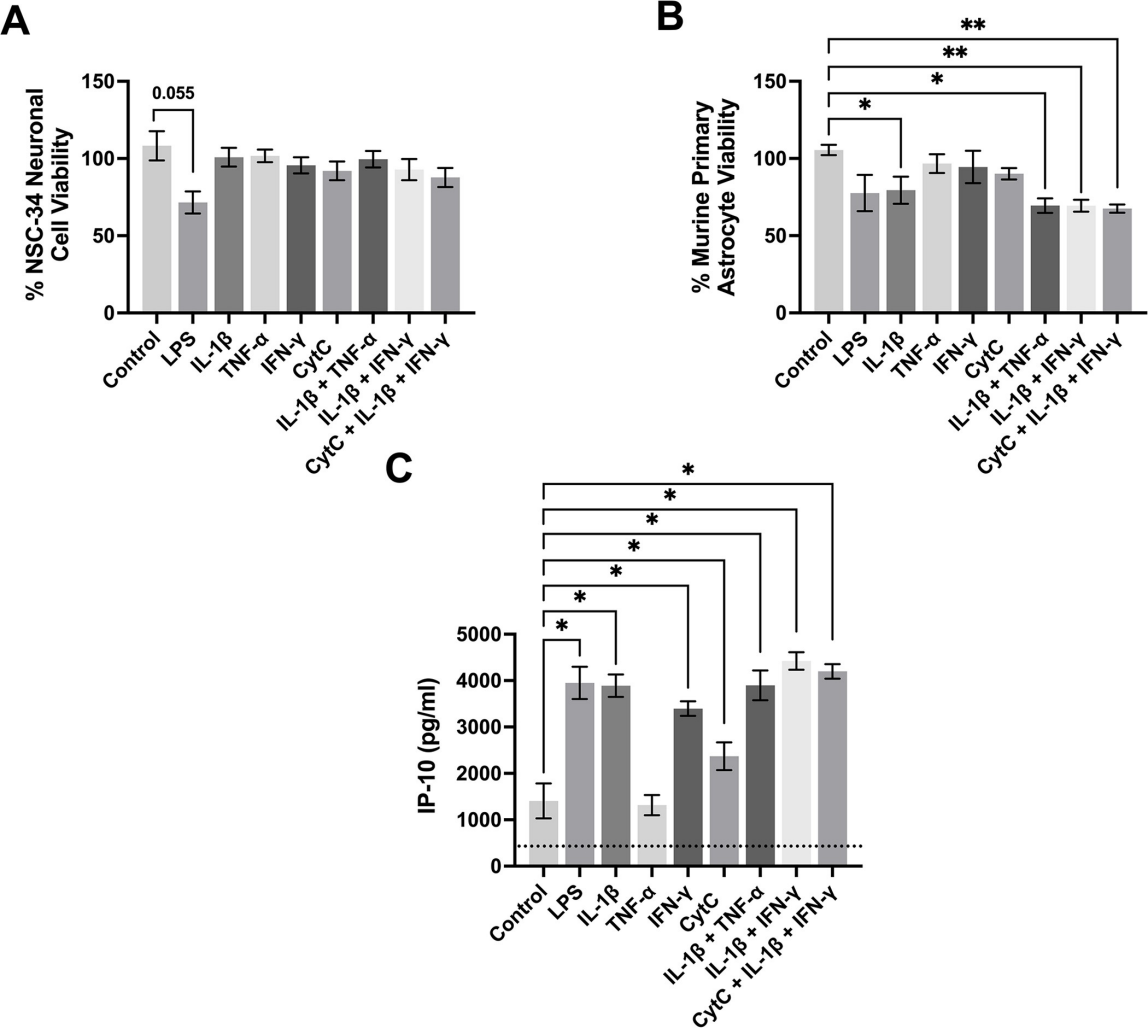
Effects of immune mediators on the toxicity of murine astrocytes towards neuronal cells and secretion of IP-10. Murine primary astrocytes were treated with the immune mediators shown on the x-axis or their vehicle solution (PBS, Control), followed by transfer of their supernatants 48 h later to murine NSC-34 neuronal cell cultures (A, B). The MTT assay was used to determine the viability of astrocytic cells (B) immediately after the transfer of supernatants. Neuronal cell viability was measured by the MTT assay after 72 h incubation with astrocytic cell supernatants (A). An ELISA was used to measure the concentration of IP-10 (C) in cell culture supernatants. Data from six (A, B) or three (C) independent experiments are presented as means ± SEM. * P < 0.05 and ** P < 0.01, different from vehicle solution-treated cells according to the paired Student’s t-test, followed by Holm-Šídák’s correction for multiple comparisons. The limit of detection for the ELISA is shown as a dotted line.

Next, we investigated the regulation of astrocyte phagocytic activity by using a murine brain-specific *in vitro* assay. Fig 4A illustrates that phagocytosis of murine brain-derived synaptosomes by murine primary astrocytes was modulated by all five inflammatory mediators used in this study. The phagocytic activity of murine cells was significantly upregulated by LPS, IL-1β, TNF-α, and CytC, as well as by all three combinations of inflammatory mediators studied (IL-1β+TNF-α, IL-1β+IFN-γ, and CytC+IL-1β+IFN-γ). IFN-γ was the only cytokine that downregulated the phagocytosis of murine synaptosomes by primary astrocytes. At the concentration tested, IFN-γ was not toxic to astrocytes (Fig 5B). It is important to note that only GFAP-positive cells were included in this analysis. Engulfment of synaptosomes by astrocytes was confirmed by combining immunocytochemistry and confocal microscopy. Fig 4B shows a representative image confirming the intracellular location of fluorescently-labeled brain-derived synaptosomes in GFAP-positive murine primary astrocytes.

### Effects of inflammatory mediators on the secretion of cytotoxins and cytokines by murine astrocytes

Fig 5A illustrates that out of all the mediators and their combinations tested, only LPS could induce toxicity of murine astrocyte supernatants towards murine NSC-34 neuronal cells, since incubation with this mediator resulted in a trend towards significant reduction of neuronal cell viability (P = 0.055). IL-1β at the concentration used, as well as all three combinations of mediators, caused a small, but significant, reduction in the viability of murine primary astrocytes (Fig 5B).

Fig 5C shows that cultured murine primary astrocytes secreted low concentrations of IP-10 in the absence of any stimuli. Nevertheless, four out of the five inflammatory mediators (except TNF-α), as well as all three combinations of inflammatory mediators tested, significantly upregulated secretion of IP-10. This chemokine, which is present in normal brains but can be upregulated in neuroinflammatory conditions such as AD, contributes to the recruitment of immune cells to the sites of inflammation and could be one of the potentially neurotoxic molecules secreted by astrocytes (reviewed in [60]) [61,62].

## Discussion

We hypothesized that the phagocytic activity of astrocytes could be modulated by the endogenous immune mediators of the CNS known to regulate microglial phagocytic activity. Therefore, we selected a subset of immune mediators, most of which have been previously reported to regulate the phagocytic activity of microglia. We also included LPS, a bacterial endotoxin, which others have shown to upregulate the phagocytic activity of not only microglia [20,63] but also astrocytes [36]. Similar to the previously reported upregulation of latex beads uptake by newborn rat forebrain astrocytes [36], LPS in our experiments enhanced the uptake of fluorescent beads by human astrocytic cells as well as murine brain synaptosomes by cultured murine primary astrocytes.

Our experiments also demonstrated that IFN-γ downregulated the phagocytic activity of both human U118 MG astrocytic cells and murine primary astrocytes. To the best of our knowledge, regulation of the phagocytic activity of astrocytes by this cytokine has not been reported before. Even though IFN-γ has been shown to modulate microglial phagocytic activity, both up- and down-regulation of this function have been observed. For example, exposure of rat primary microglia to IFN-γ for four hours increased areas of axonal debris clearance [31]. In contrast, 24 h incubation with IFN-γ decreased engulfment of beads by murine MMGT12 microglial cells and by murine primary microglia [32,64].

The other two cytokines used in this study, IL-1β and TNF-α, upregulated phagocytosis of synaptosomes by murine primary astrocytes, but had no effect on the phagocytic activity of human astrocytic cells. The effects of these cytokines on astrocyte phagocytic activity have not been studied before; however, it should be noted that rat cortical astrocytes exposed to TNF-α, as well as IFN-γ, demonstrated enhanced myelin uptake, which was determined to be mediated by endocytic mechanisms [29]. Nevertheless, both IL-1β and TNF-α are known to upregulate the phagocytic activity of microglia. For example, IL-1β enhanced phagocytosis of opsonized latex beads by N9 murine microglial cells [63], while TNF-α increased phagocytosis of beads and live neurons by murine primary microglia [42]. Overall, our observations indicate that the phagocytic activity of the two main glial cell types, astrocytes and microglia, could be regulated similarly. Our study also identifies possible differences in the regulation of phagocytic activity of human U118 MG astrocytic cells and murine primary astrocytes. This observation will require further studies since this disparity in our experiments could be due to not only different species, but also types of cells used (immortal cell line vs. primary cultured cells) as well as variance in engulfed elements (latex beads vs. murine synaptosomes). Future experiments using primary cells should characterize the regulation of phagocytic activity of human astrocytes since this function is believed to be impaired in several disorders, including Alzheimer’s diseases and multiple sclerosis (reviewed in [65]) [33].

In addition to the established endogenous regulators of phagocytosis, we also tested CytC, a mitochondria-derived DAMP (reviewed in [66,67]), as a possible novel regulator of astrocyte phagocytic activity. To the best of our knowledge, the effect of CytC on phagocytosis has not been studied in any cell type. In this study, we demonstrated for the first time that CytC enhanced the phagocytic activities of both human astrocytic cells and murine primary astrocytes, adding to the previously reported CytC-induced upregulation of astrocyte secretion of cytokines and cytotoxins [44].

Several previous studies have demonstrated synergistic effects of inflammatory mediators on diverse functions of astrocytes. The importance of studying combinations of stimuli on neuroimmune functions of these cells is becoming increasingly evident, since in many cases, astrocytes respond to such combinations only and not the individual cytokines or other inflammatory mediators [e.g., 45–47]. We chose to study three different combinations of inflammatory mediators: IL-1β plus TNF-α, IL-1β plus IFN-γ, and CytC in combination with IL-1β and IFN-γ. Human astrocyte secretome profiling has demonstrated that the combination of IL-1β plus TNF-α upregulates secretion of multiple cytokines [11]. IL-1β plus IFN-γ have been shown to induce reactive oxygen species production, NO and TNF-α secretion, trigger neurotoxicity, and downregulate glutamate uptake in human astrocytes [68]. Since CytC has been shown to engage human astrocyte TLR4 [44], adding this endogenous ligand to the combination of IL-1β plus IFN-γ, which act on their own receptors, was hypothesized to enhance the proinflammatory potential of the cytokine mixture. Our experiments assessing the effects of these three combinations of inflammatory mediators on astrocyte phagocytic activity identified several unique trends. In human U118 MG astrocytic cells, IFN-γ alone and its combinations with IL-1β and CytC downregulated phagocytic activity, but the combination of IL-1β plus TNF-α had no effect, similar to individual application of these cytokines. In murine primary astrocytes, the IL-1β and TNF-α combination upregulated phagocytic activity, which was consistent with the effects of individual applications of these cytokines, but no synergistic effect was observed. Dissimilar to human astrocytic cells, the two combinations containing IFN-γ upregulated phagocytic activity of murine astrocytes, likely due to the presence of other mediators (IL-1β and CytC) that had a more potent stimulatory effect on this function of astrocytes.

Murine astrocytes express TLR4 *in vitro* in both mixed and purified cultures [69], and functional TLR4 has also been demonstrated in human primary astrocytes [44,70]. Both LPS and CytC have been shown to modulate other functions of astrocytes, such as inducing the secretion of proinflammatory cytokines, by interacting with TLR4 [44,71], and ligands of this receptor regulate the phagocytic activity of microglia and other mononuclear phagocytes [72–74]. Therefore, we first hypothesized and then demonstrated by using a specific inhibitor, TAK-242, that both LPS- and CytC-induced phagocytic activity of human astrocytic cells was at least partially mediated by TLR4. Further studies will be needed to decipher signaling pathways that are activated by CytC interacting with astrocytic TLR4; however, previous research has demonstrated that phagocytic activity in diverse cell types is upregulated by activation of both myeloid differentiation primary-response protein 88 (MyD88)-dependent and MyD88-independent pathways that are linked to TLR4 (reviewed in [75]). Furthermore, LPS-induced and TLR4-dependent phagocytosis by microglia has been shown to require activation of nuclear factor (NF)-κB [76]. In our study, IFN-γ was the only mediator that downregulated phagocytic activity in both human and murine cells. It has already been demonstrated in murine macrophages that IFN-γ inhibits phagocytosis of non-opsonized particles by engaging the phosphatidylinositol 3-kinase (PI3K)-Akt pathway, inducing mammalian target of rapamycin (mTOR), and decreasing the expression of CCAAT enhancer-binding protein β (c/EBPβ) [77]. The role of this pathway in IFN-γ-induced downregulation of astrocyte phagocytic activity will require experimental proof.

We were also interested to find out whether the pattern of modulation of the phagocytic function of astrocytes by various mediators corresponds to that of their effects on other neuroimmune functions of this cell type that have been studied by us and others before [41,57,78,79]. We examined the secretion of select inflammatory cytokines, glutamate release, and overall cytotoxicity towards neuronal cells after exposing astrocytic cells to the various inflammatory mediators and their combinations. Overall, no correlations could be identified between the effects of these mediators on phagocytic activity and other functions of astrocytes. In contrast to the modulation of phagocytic activity in human U118 MG astrocytic cells by LPS, CytC, and IFN-γ, only LPS was able to induce secretion of cytokine GM-CSF and toxins capable of killing human SH-SY5Y neuronal cells. We ruled out NO as a potential cytotoxin responsible for the toxicity of LPS-stimulated U118 MG cells towards SH-SY5Y cells by demonstrating that LPS did not induce significant release of NO by U118 MG cells under the experimental conditions of this study. Our previous study showed that LPS under the same experimental conditions did not induce secretion of another potentially neurotoxic cytokine, IL-1β [41].

Furthermore, supernatants from murine astrocytes exposed to LPS, but none of the other mediators, showed a trend towards toxic effects when applied to murine NSC-34 neuronal cells, which again was different from the regulation of phagocytic activity by all mediators tested in this study. Interestingly, secretion of IP-10 by murine astrocytes was significantly upregulated by all mediators, and their combinations studied, except TNF-α on its own. Notably, upregulation of IP-10 secretion in murine astrocytes by IFN-γ was opposite to its effects on their phagocytic activity. It can be concluded that distinct mechanisms likely regulate astrocyte phagocytic activity and their secretion of cytokines, cytotoxins, and glutamate [79]. Our observation of LPS-induced IP-10 secretion by murine primary astrocytes is consistent with a previous report that LPS enhances IP-10 mRNA expression in murine cultured astrocytes [80]. In addition, Bakmiwewa et al. [46] used murine astrocytic C8D1A cells to demonstrate a synergistic effect of IFN-γ and lymphotoxin-α on IP-10 mRNA expression and protein secretion. The aim of our study was to assess the regulation of only select cytokines by inflammatory mediators; therefore, we did not study regulation of a broad range of cytokines by astrocytic cells. Even though there was no correlation between regulation of phagocytic activity and secretion of IP-10 by murine astrocytes, we cannot rule out that secretion of other astrocyte cytokines such as IL-6, IL-8, and TNF-α may be modulated similar to their phagocytic activity. Furthermore, since only single concentrations of the mediators were tested, it is possible that using different concentrations of the same stimuli would lead to alternative patterns of changes in astrocytic cell functions. It should be noted that overall, the chosen concentrations of mediators led to satisfactory experimental outcomes since all mediators induced significant changes in at least one of the cell activation parameters studied. In addition, none of the mediators or their combinations at the concentrations tested caused toxic effects towards human U118 MG astrocytic cells while only IL-1β and the combinations of mediators induced small, but significant, reduction in viability of primary murine astrocytes.

Several of the inflammatory mediators used in this study upregulated astrocyte phagocytic activity without inducing their secretion of cytotoxins; however, the transfer of astrocytic cell supernatants onto neuronal cells used in our experiments did not allow assessing cell contact-dependent, phagocytosis-mediated toxicity, such as phagoptosis that has been described for microglia (reviewed in [81]). Even though astrocyte-mediated phagoptosis has not been reported before, this potential function of astrocytes should be explored in future studies using astrocyte-neuron co-culture models.

## Conclusion

Several previous studies have documented the phagocytic activity of astrocytes [24,34,41]; however, the shortage of information about the endogenous regulators of this critical function of astrocytes was the knowledge gap addressed by our current research. By using an inhibitor of actin polymerization, we first confirmed that U118 MG cells phagocytose latex beads and employed these cells as models of human astrocytes, in addition to using murine primary astrocytes. We also investigated phagocytosis of synaptosomes by murine primary astrocytes. Our experiments identified IL-1β, TNF-α, IFN-γ, and CytC as possible endogenous to the CNS regulators of astrocyte phagocytic activity. We also confirmed a previous report that this astrocytic function can be regulated by the bacterial endotoxin LPS [36]. Our data also identified TLR4 as one of the receptors modulating the phagocytic function of astrocytes. Recent studies indicate that phagocytic activity of astrocytes is not only essential during development and under physiological CNS conditions, but that its dysregulation could contribute to multiple neuropathologies, such as demyelination after brain ischemia, multiple sclerosis, and neurodegenerative diseases (reviewed in [65,82,83]) [13,34]. Therefore, further in-depth studies of the receptors and intracellular signaling pathways regulating phagocytic function in astrocytes are warranted. Such research could identify novel therapeutic targets for restoring or adjusting this astrocyte-mediated clearance mechanism.

## Supporting information

S1 Fig Effects of immune mediators on the NO secretion by U118 MG astrocytic cells. (PDF)

S2 Fig Effects of immune mediators on the human astrocytic cell secretion of IL-6 and IP-10. (PDF)

S1 Table Raw data. (XLSX)
