# Supplementary material for: Regulation of the phagocytic activity of astrocytes by neuroimmune mediators endogenous to the central nervous system
Source: PLoS One. 2023 Jul 27;18(7):e0289169. doi: 10.1371/journal.pone.0289169 (PMC10374099; doi:10.1371/journal.pone.0289169)
Supplement: S1 Fig — (PDF) [file pone.0289169.s001.pdf]

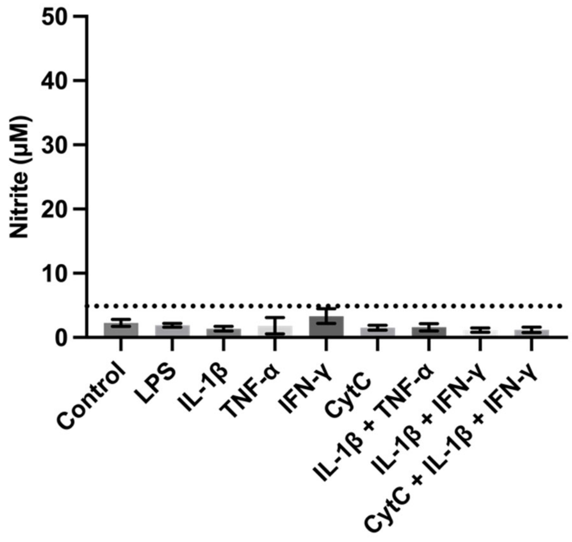

S1 Fig. Effects of immune mediators on the NO secretion by U118 MG astrocytic cells.

Human U118 MG astrocytic cells were treated with the immune mediators shown on the x-axis or their vehicle solution (PBS, Control) for 48 h. A Griess assay was used to measure the NO secreted by cells [43]. Data from four independent experiments are presented as means ± SEM. The limit of detection for the Griess assay is shown as dotted line.
