# Supplementary material for: Regulation of the phagocytic activity of astrocytes by neuroimmune mediators endogenous to the central nervous system
Source: PLoS One. 2023 Jul 27;18(7):e0289169. doi: 10.1371/journal.pone.0289169 (PMC10374099; doi:10.1371/journal.pone.0289169)
Supplement: S2 Fig — (PDF) [file pone.0289169.s002.pdf]

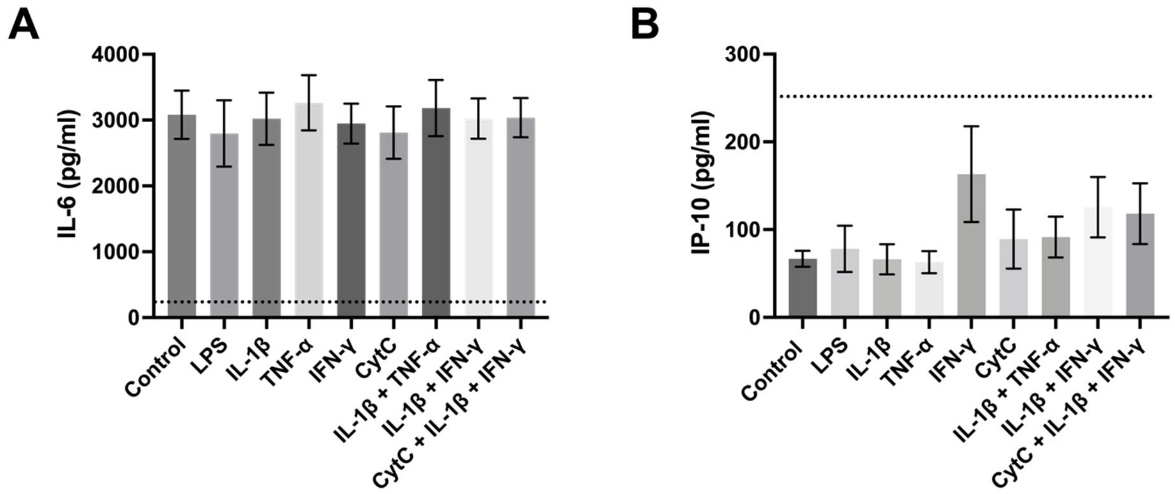

S2 Fig. Effects of immune mediators on the human astrocytic cell secretion of IL-6 and IP-10.

Human U118 MG astrocytic cells were treated with the immune mediators shown on the x-axis or their vehicle solution (PBS, Control) for 48 h. An ELISA was used to measure the concentration of IL-6 (A) and IP-10 (B) in cell culture supernatants. Data from four (A) or five (B) independent experiments are presented as means ± SEM. The data were analyzed using paired Student's t-test, followed by Holm-Šídák's correction for multiple comparisons. The limits of detection for the ELISAs are shown as dotted lines.
